# Hidden in the woods: A new species of *Hexura* (Araneae, Mygalomorphae, Antrodiaetidae) from a highly restricted range in Idaho

**DOI:** 10.3897/zookeys.1290.200898

**Published:** 2026-08-31

**Authors:** Arnau Calatayud-Mascarell, Ethan J. Briggs, Chris A. Hamilton

**Affiliations:** 1 Department of Entomology, Plant Pathology and Nematology, University of Idaho, Moscow, ID, 83844 USA Department of Entomology, Plant Pathology and Nematology, University of Idaho Moscow United States of America; 2 Entomology & Arachnology, Australian Museum Research Institute, Australian Museum, 1 William St, Darlinghurst NSW 2010, Sydney, Australia Entomology & Arachnology, Australian Museum Research Institute, Australian Museum Sydney Australia

**Keywords:** Micro-endemism, Pacific Northwest, Rocky Mountains, spiders, taxonomy, Ultraconserved elements

## Abstract

A new species of *Hexura* Simon, 1885 is described from a very restricted geographic range in the Rocky Mountains of Idaho, 500 km east of the previously known distribution of the genus in the Coastal Ranges and western Cascade Mountains of Oregon and Washington. This work provides the first taxonomic information for this group in almost 50 years, including a phylogenomic framework for the genus within the mygalomorph superfamily Atypoidea, based on Ultraconserved elements (UCEs). *Hexura vandal***sp. nov**. can be distinguished from the other two described species by a combination of morphological, molecular, and geographic characteristics. Lastly, we discuss the need for conservation actions to secure the survival of the species.

## Introduction

**Figure 1.**
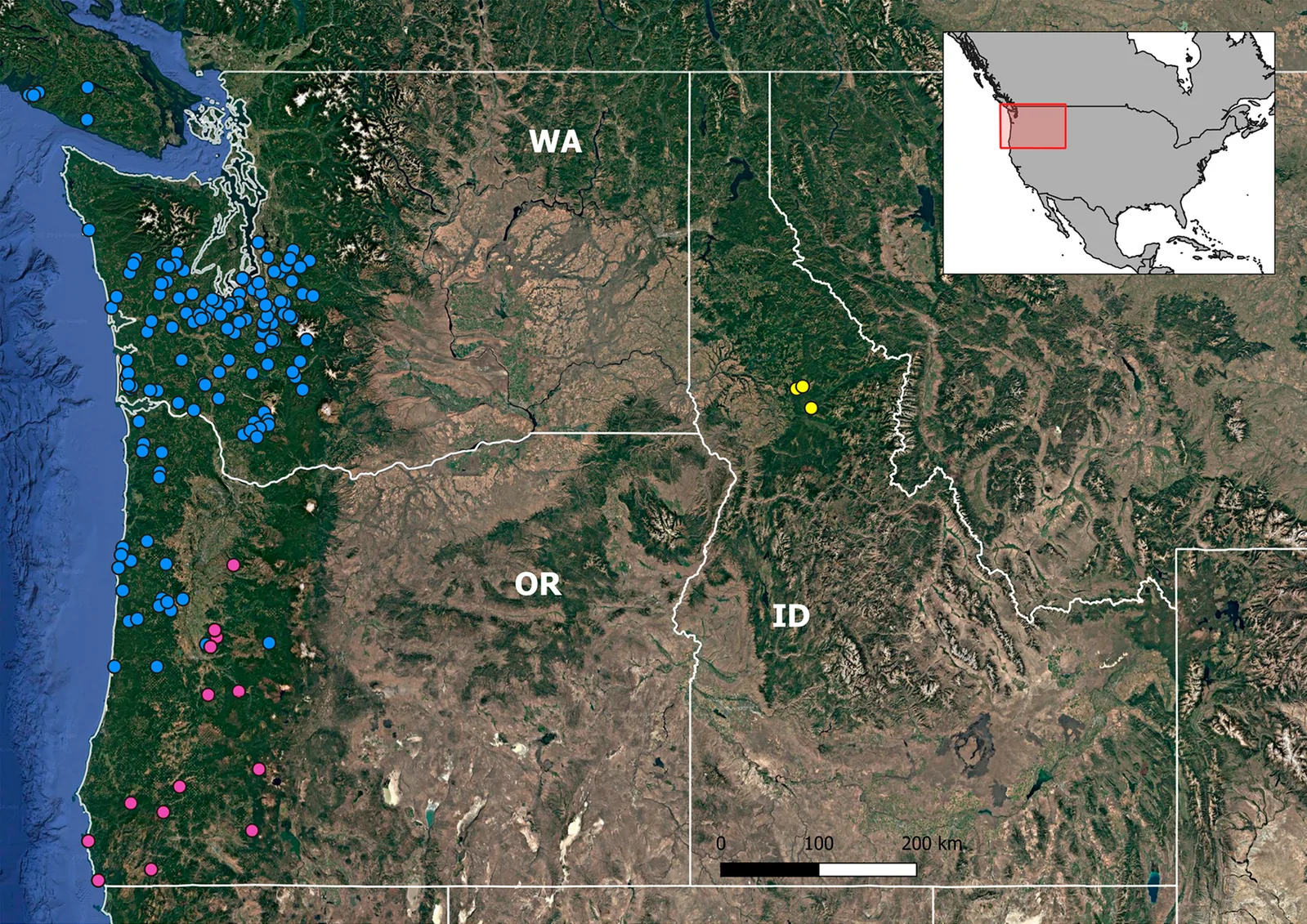
Distribution and habitat of *Hexura* spiders including new records, records from iNaturalist, Gertsch and Platnick (1979), and The Burke Museum of Natural History in Seattle, Washington. Blue = *H. picea*, Pink = *H. rothi*, Yellow = *H. vandal*.

**Figure 2.**
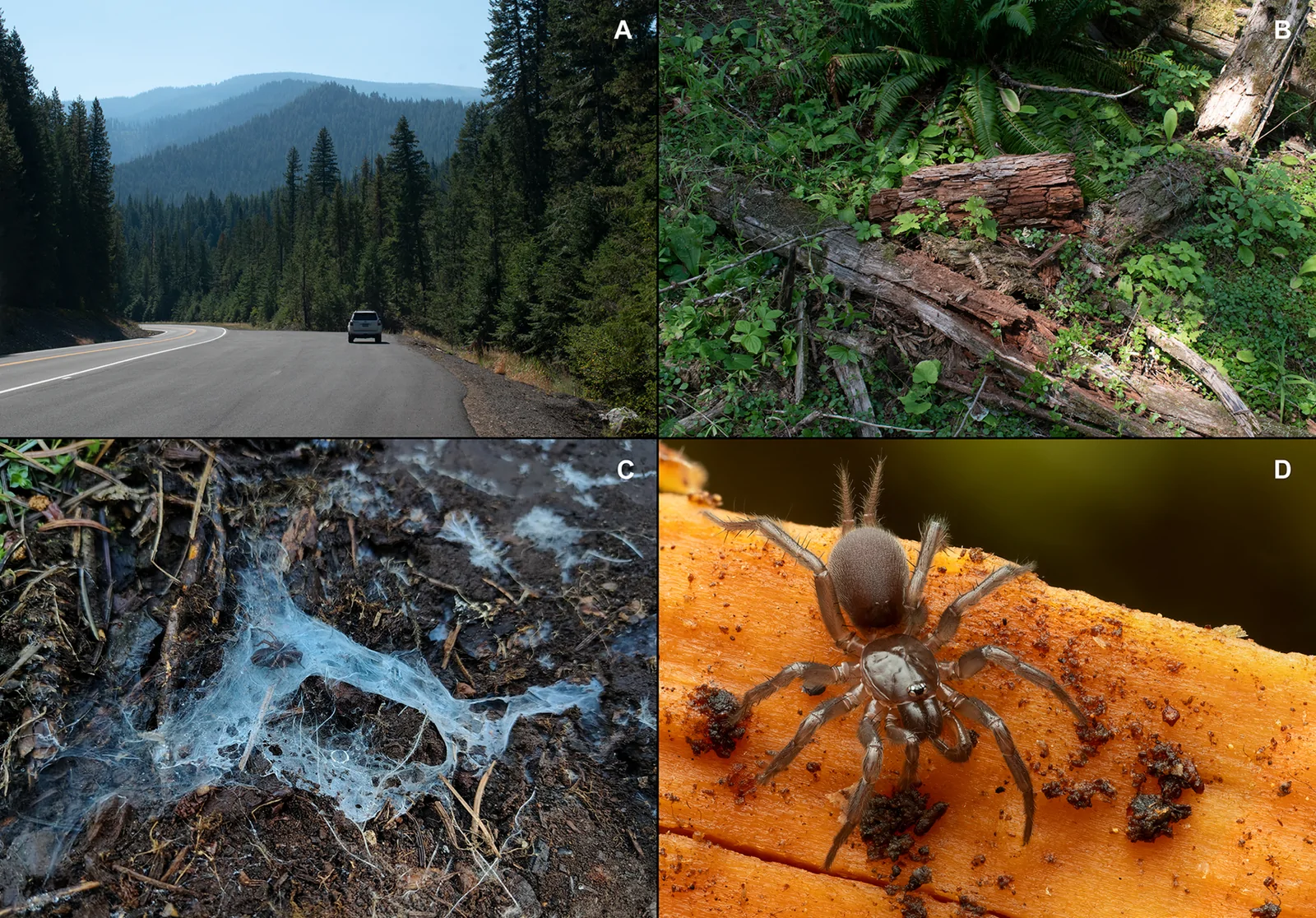
*Hexura* habitat photographs. **A**. US Highway 12, near *Hexura vandal* sp. nov. type locality, Apgar Campgrounds, Lowell, Idaho; **B**. Mixed conifer forest logs, *Hexura vandal* sp. nov. type locality, Apgar Campgrounds, Lowell, Idaho; **C**. *Hexura rothi* funnel-web, Eagle Point, Oregon (photo credit Cesar Garcia Olmos); **D**. *Hexura picea*, Capital Regional District, BC, Canada (photo credit Thomas Barbin).

The genus *Hexura* Simon, 1885 includes two species of mygalomorph funnel-web spiders endemic to North America’s Pacific Northwest (PNW) Coastal Ranges and Cascade Mountains (Gertsch and Platnick 1979). The northern species, *Hexura picea* Simon, 1885 ranges from southern Vancouver Island to central Oregon, while the southern species, *Hexura rothi* Gertsch & Platnick, 1979 has a smaller distribution, from central to southern Oregon (Fig. 1). These spiders inhabit mesic conifer forests and exhibit an opportunistic lifestyle, building their silken funnel-web retreats under or inside logs, or in the moss mat microhabitats of these forests (Gertsch and Platnick 1979; personal observation) (Fig. 2). Although their distribution overlaps in central Oregon, they can be easily distinguished by the number of spinnerets: *H. picea* has three pairs, while *H. rothi* has only two. The first and only taxonomic revision of the genus by Gertsch and Platnick (1979) placed *Hexura* within the atypoid family Mecicobothriidae. However, Mecicobothriidae was found to be non-monophyletic after a phylogenomic investigation of atypoid mygalomorphs; *Hexura* was transferred into the family Antrodiaetidae (Hedin et al. 2019). Divergence estimates found an ancient origin and split of *Hexura* species (60–180 Ma), suggesting that the group represents a relictual lineage with a deep evolutionary history, whose present-day distributions may reflect ancient biogeographic events rather than recent dispersal (Hedin et al. 2019).

Mesic conifer forests that were once connected throughout the PNW split after the formation and progressive xerification of the Columbia Basin, from the late Miocene to early Pliocene (Brunsfeld et al. 2001). Today, the Columbia Basin acts as a significant barrier to mesic-adapted plant and animal species distributed across the PNW (Graham 1999). Several biogeographic hypotheses have been proposed for low-dispersal taxa in the PNW that are susceptible to vicariance, with debate focusing on whether divergence between Cascade and Rocky Mountain populations reflects recent or ancient vicariant events (Carstens et al. 2005; Richart and Hedin 2013; Ruffley et al. 2022). Interestingly, while other ecologically similar mygalomorph spiders such as *Antrodiaetus* Ausserer, 1871 (Ciaccio et al. 2024) and *Microhexura* Crosby & Bishop, 1925 (Coyle 1981) were known from both the Cascade and Rocky Mountains, *Hexura* had not been found in the Rocky Mountains.

One hundred and fifty years after the discovery of *Hexura*, we discovered a novel lineage of *Hexura* from the Rocky Mountains in northern Idaho. These new geographical records extend the distribution of the genus about 500 km from its original distribution. We provide a phylogenomic placement of this novel species within atypoid mygalomorph spiders using Ultraconserved elements (UCEs). By integrating molecular, morphological, and geographic data, we describe *Hexura vandal* sp. nov., revise the genus, and provide new insights into the evolutionary history of *Hexura*. *Hexura vandal* sp. nov. is only found in an extremely restricted distribution within Idaho. This finding, combined with its ancient evolutionary history and the ongoing biodiversity crisis, highlights the need to recognize this unique lineage and establishes the foundation for potential conservation measures to secure the future of the species.

## Material and methods

### Sampling

Fifty-three samples were collected for morphological examination; this includes *H. picea* (9), *H. rothi* (3) and *H. vandal* sp. nov. (41) (see Suppl. material 2). Additional material from the American Museum of Natural History (AMNH) was examined, including 29 *H. rothi* and 18 *H. picea* (see Suppl. material 2). The type material for *H. picea* from “Washington-Territory” was deposited in the Muséum national d’Histoire naturelle, Paris, France (Gertsch and Platnick 1979), though it was not examined. Even though the holotype was not examined, no other mygalomorph from Washington has three spinnerets, thus, the examination of the holotype is not essential for a confident identification as *H. picea*. For phylogenetic inference, we gathered 26 samples from 14 species representing all the families within Atypoidea, including newly sequenced material and material available on the Sequence Read Archive (SRA) database generated from previous studies (Starrett et al. 2017; Hedin et al. 2019; Ciaccio et al. 2024; Monjaraz‐Ruedas et al. 2024) (see Suppl. material 2).

### DNA extraction and sequencing

To generate a phylogenomic dataset, Ultraconserved elements (UCEs) were chosen because they represent a genome-wide sampling technique that can provide resolution at both deep and shallow nodes within the Tree of Life (Faircloth et al. 2012), making them a particularly useful tool for studying lineages like *Hexura* and the superfamily Atypoidea, which has a deep evolutionary history. We extracted genomic DNA from ethanol-preserved spider specimens using Qiagen QIAamp Micro DNA purification kit (Qiagen, Valencia, CA). DNA was quantified on the Qubit 4 Fluorometer (Thermo Fisher), and DNA length and integrity were assessed by 1% agarose gels run at 120 V for 30 min. DNA samples were stored at 4 °C prior to library preparation. Library preparation used the SRSLY NanoPlus Uracil+ Base Kit (Troll et al. 2019) and UDI Primers from ClaretBio (Santa Cruz, CA). DNA was sheared to a mean fragment length of 300 bp using ClaretBio ForShear Enzymatic Shearing Module, with fragments then undergoing adapter ligation, indexing PCR, and library purification with ClaretBio Clarefy DNA purification beads. UCE loci were captured using the Spider 2K v. 1 probeset kit from Arbor Biosciences. Enriched libraries underwent amplification (Roche KAPA HiFi readymix with IDT xGen Library Amplification primers) and purification using ClaretBio Clarefy DNA purification beads, before being sent to NovoGene (Sacramento, CA) for sequencing. DNA was sequenced on NovaSeq X Plus Series PE150.

### Data processing

The raw sequencing data were processed using Phyluce v. 1.7.1 (Faircloth 2016) and a combined arachnid-spider probe set file (Maddison et al. 2020), merging the original Arachnida probe set (Starrett et al. 2017) with the Spider 2K v. 1 probe set (Kulkarni et al. 2020), using match settings of 65 for minimum identity and minimum coverage. Loci were aligned using MAFFT (Katoh and Standley 2013), with alignments externally trimmed to clean poorly aligned edges and then internally trimmed using gBlocks (Castresana 2000) to remove poorly aligned blocks within the sequences under the following settings: b1 = 0.5, b2 = 0.5, b3 = 6, and b4 = 6. A 60% occupancy matrix (i.e., concatenated dataset) was generated for subsequent analyses, as this was a balance between completeness and the number of quality loci. Data were further cleaned using Spruceup (Borowiec 2019), which removes poorly aligned sequence fragments from individual sequences within alignments. A visual inspection of the distance distribution plots indicated that a setting of 0.9 was optimal for this trimming.

### Phylogenomic analyses

A maximum likelihood-based phylogeny was inferred using IQ-TREE 2 (Minh et al. 2020) on the FortyFive cluster within the Research Computing and Data Services (RCDS) center at the University of Idaho. Support values were assessed using 1000 ultrafast bootstrap replicates and 1000 replicates of the SH-like Approximate Likelihood Ratio Test (SH-ALRT). The concatenated data were partitioned by UCE loci, with optimal models of evolution for each partition estimated using MODELFINDER (Kalyaanamoorthy et al. 2017), a program integrated into IQ-TREE 2. A species tree was inferred using ASTRAL-III (Zhang et al. 2018) under a heuristic quartets sampling approach that incorporated the individual gene trees inferred from the 60% occupancy matrix using IQ-TREE 2 with 1000 bootstrap replicates.

### Taxonomy and morphological analysis

Linear measurements of 27 somatic and two male sexual characters were taken from 27 specimens, including five males and five females of *H. picea*, five males and five females of *H. rothi* and two males and five females of *H. vandal* sp. nov. Detailed information on the measured specimens and measurements can be found in the Suppl. material 2 (Morphometrics dataset). The abbreviations for the measured morphological characters are: carapace length (Cl), carapace width (Cw), labium length (Ll), labium width (Lw), femur I length (F1l), femur I width (F1w), patella I length (P1), tibia I length (Ti1), metatarsus I length (M1), tarsus I length (Ta1), femur II length (F2l), femur II width (F2w), patella II length (P2), tibia II length (Ti2), metatarsus II length (M2), tarsus II length (Ta2), femur III length (F3l), femur III width (F3w), patella III length (P3), tibia III length (Ti3), metatarsus III length (M3), tarsus III length (Ta3), femur IV length (F4l), femur IV width (F4w), patella IV length (P4), tibia IV length (Ti4), metatarsus IV length (M4), tarsus IV length (Ta4), palpal tibia length (PTl), and palpal tibia width (PTw).

Measurements were reported in millimeters, rounded to two digits, and were taken from left appendages using a Leica M205 C stereomicroscope. Specimens were digitally imaged with Capture One 12 software using a Canon EOS 5DS R with a 65 mm 1×–5× Macro lens. Individual images were combined (i.e., stacked) into a composite image using Zerene Stacker v. 1.04 and edited with Adobe Photoshop. Specimens were placed on KY Jelly lubricant immersed in 80% ethanol to secure the targeted position. Female spermathecae were dissected using forceps and fine entomological pins. After extraction, spermathecae were cleaned in a centrifuge tube with Trypsin 1:75 powder and distilled water. The tube was incubated for 24 h in a Thermomixer at 34 °C and 400 rpm. The spermathecae were further cleaned manually with fine entomological pins and washed with 80% ethanol. Spermathecae images were taken as above but used a Canon EF 200 mm lens attached to an M Plan Apo 10× objective.

The morphospace occupied by each lineage was examined by plotting measurements in a Principal Component Analysis (PCA), Non-metric Multidimensional Scaling (NMDS) analysis and a Canonical Variate Analysis (CVA). PCA analyses and graphics were made in R using “FactoMineR” (Lê et al. 2008), “factoextra” (Kassambara and Mundt 2020) and “ggplot2” (Wickham 2016) packages. NMDS analyses and graphics were made in R using “vegan” (Dixon 2003) and “ggplot2” (Wickham 2016) packages. CVA analyses and graphics were made in R using the first 5 PCs with the package “ggplot2” (Wickham 2016). The male holotype and female paratypes of *H. vandal* sp. nov. have been deposited in the Arthropod Molecular Systematics Lab, part of the University of Idaho William F. Barr Entomological Museum, Moscow, Idaho.

## Results and discussion

### Phylogenomic analyses

**Figure 3.**
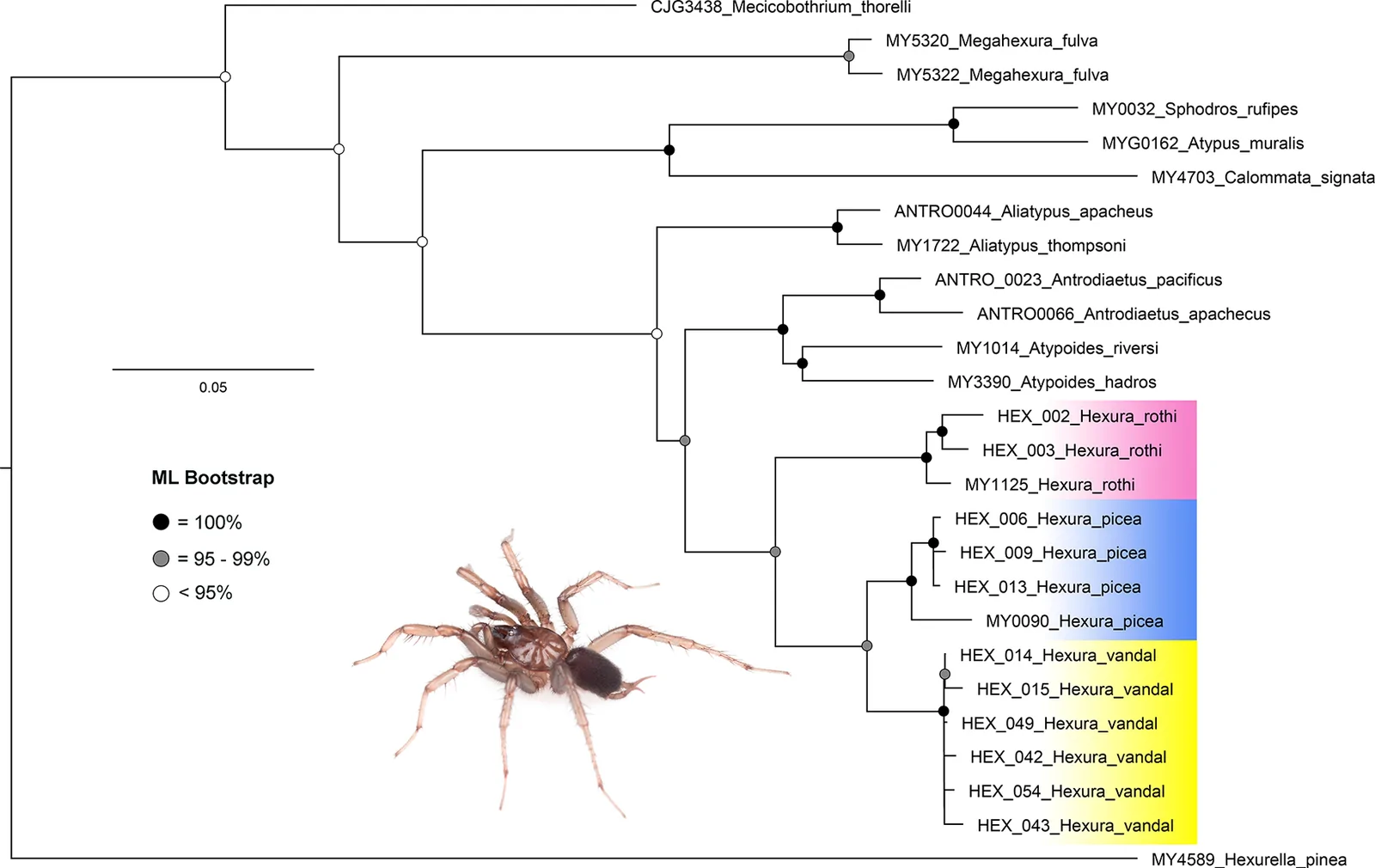
Molecular phylogeny of the mygalomorph superfamily Atypoidea using an Ultraconserved element concatenated supermatrix under a maximum likelihood approach with IQ-TREE 2.

With a dataset consisting of 26 specimens, 728 loci were obtained from a 60% occupancy matrix for a total of 292,837 base pairs. Raw reads can be found on the Sequence Read Archive (PRJNA1458865). Results are consistent with the phylogenomic analysis of Atypoidea (Hedin et al. 2019), recovering the same topology and similar support values (Fig. 3). The genus *Hexura* was recovered within the family Antrodiaetidae, sister to the *Antrodiaetus* and *Atypoides* clade. All three *Hexura* species were recovered as monophyletic with ufBS of 100. *Hexura vandal* sp. nov. was recovered as sister to *H. picea* (ufBS = 99), with *H. rothi* recovered as sister to the other two species (ufBS = 99). The species tree with ASTRAL-III also supports the three species hypothesis with the same relationships within *Hexura* (local posterior probability = 1) (Suppl. material 1: fig. S1).

As predicted by the number of spinnerets, *Hexura vandal* sp. nov. is sister to *H. picea*, a result that indicates three pairs of spinnerets may be a derived character for the genus, with two pairs of spinnerets the ancestral character retained in *H. rothi*. Future research should investigate whole-genome data to better understand the evolution of spinneret morphology in Atypoidea. Generating reference genomes for *Hexura* would enable comparative analyses to identify genes and regulatory elements associated with spinneret number, while transcriptomic and population-level data could clarify the developmental mechanisms and evolutionary history underlying the transition from two to three pairs.

The discovery of *H. vandal* sp. nov. in the northern Idaho Rocky Mountains substantially extends the known distribution of the genus by approximately 500 km, representing a significant range disjunction within the PNW, changing the fundamental understanding of these spiders and their biogeographic history. Such findings are particularly informative for mygalomorph spiders, whose habitat fidelity, cryptic lifestyle, limited dispersal abilities, and long-life span make them excellent models for historical biogeography and vicariance (e.g., Hedin et al. 2013; Opatova and Arnedo 2014; Calatayud‐Mascarell et al. 2022). Comparable cases highlight how new locality records can change our understanding of mygalomorph lineage distributions, like *Hadronyche anzses* Raven, 2000, which extended the known range of Australian atracids by 1200 km (Raven 2000), or the recent documentation of *Idioctis parilarilao* Yu, Lo, Cheng, Raven & Kuntner, 2023 in Taiwan that revealed an unexpected distributional breadth in barychelids (Yu et al. 2023). In North America, the apparent geographic and potentially evolutionary separation between *Microhexura montivaga* Crosby & Bishop, 1925 of the Appalachian Mountains of the eastern United States and *M. idahoana* Chamberlin & Ivie, 1945 of the PNW shows how isolated montane systems may promote divergence within range-restricted taxa with narrow ecological requirements (Coyle 1981). Together, these cases underscore that newly discovered, geographically distant populations are not just range extensions, but can challenge existing assumptions about how habitats were connected in the past, speciation processes, and biogeographic history.

### Morphological analyses

Although morphospace plots suggest subtle tendencies of divergence, the morphometric analyses of all measured characters do not reveal distinct clustering among females of any species (Suppl. material 1: fig. S2A, C, E). Similarly, for males, PCA and NMDS show no distinct clustering of any species, although there are subtle tendencies towards morphospace separation (Suppl. material 1: fig. S2B, D). In contrast, CVA, which maximizes discrimination among predefined groups, clearly discriminates the three species, with each forming a distinct cluster in morphospace (Suppl. material 1: fig. S2F).

### Taxonomy

#### Family Antrodiaetidae Gertsch, 1940

**Genus *Hexura* Simon, 1885**

##### Hexura picea

Taxon classificationAnimaliaAraneaeAntrodiaetidae

Simon, 1885

1AD88752-8766-5E13-979E-EF9BCEFB30BF

Figs 4, 5, 6

Hexura picea Simon, 1885: 316 (male and female syntypes from “Washington-Territory”, deposited in the Muséum national d’Histoire naturelle, Paris, France [not examined]); Simon 1903: 971, figs 1110–1111; Chamberlin and Ivie 1945: 550, figs 3–5; Gertsch and Platnick 1979: 25, figs 3, 31, 58–63, 67.

###### Material examined.

USA – Oregon, Benton Co: • 1 ♂ (AMNH_IZC 00480903), Marys Peak, 8.iv.1951, V. Roth leg.; • 1 ♂, 3 ♀ (AMNH_IZC 00480908), Marys Peak, 9.v.1985, K. W. Radtke leg. Washington, Cowlitz Co: • 2 juv. (HEX_012, HEX_013), Ape Cave Interpretative Site Trail (46.1105, −122.2117), 653 m, 16.viii.2024, A. Calatayud and E. Briggs leg. King Co: • 2 ♀, 3 juv. (AMNH_IZC 00480902), Renton, 30.iv.1936, Exline leg. Pierce Co: • 1 ♀, 1 juv. (AMNH_IZC 00480906), near Mt Rainier, 6.viii.1929, N. Chamberlin leg. Skamania Co: • 1 ♂, 2 juv. (HEX_004–006), Lake Merwin Mountain, site 1 (45.9940, −122.4347), 412 m, 15.viii.2024, A. Calatayud and E. Briggs leg.; • 2 ♂, 3 juv. (HEX_007–011), Lake Merwin Mountain, site 2 (46.0230, −122.4260), 815 m, 15.viii.2024, A. Calatayud and E. Briggs leg. Thurston Co: • 2 juv. (AMNH_IZC 00480907), Olympia, 27.xii.1931, Exline leg.; • 1 ♀ (AMNH_IZC 00480904), Olympia, 31.iv.1932, Exline leg.; • 1 ♀ (AMNH_IZC 00480905), Olympia, vi.1944, Exline leg.

**Figure 4.**
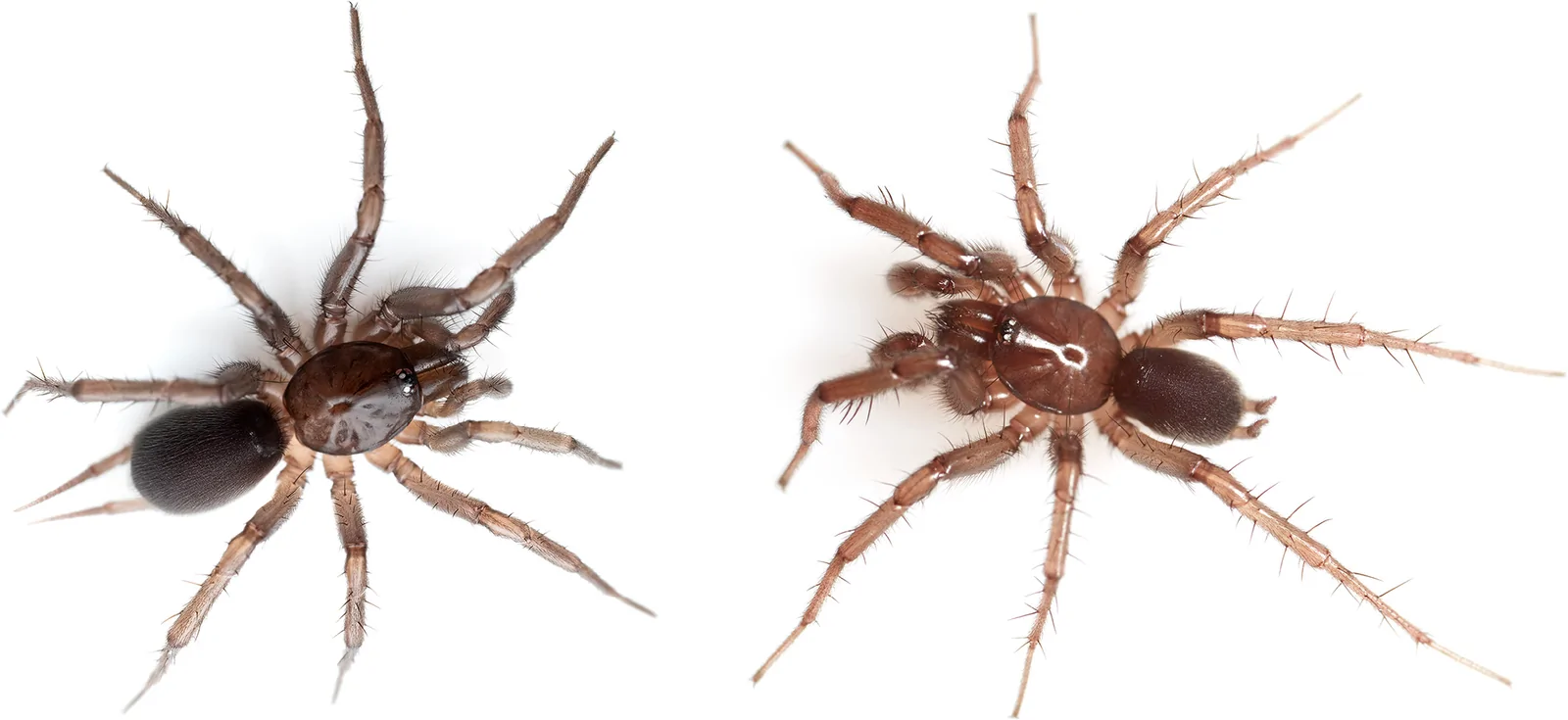
*Hexura picea* female (L) (photo credits to Thomas Barbin), male (R).

###### Diagnosis.

**Figure 5.**
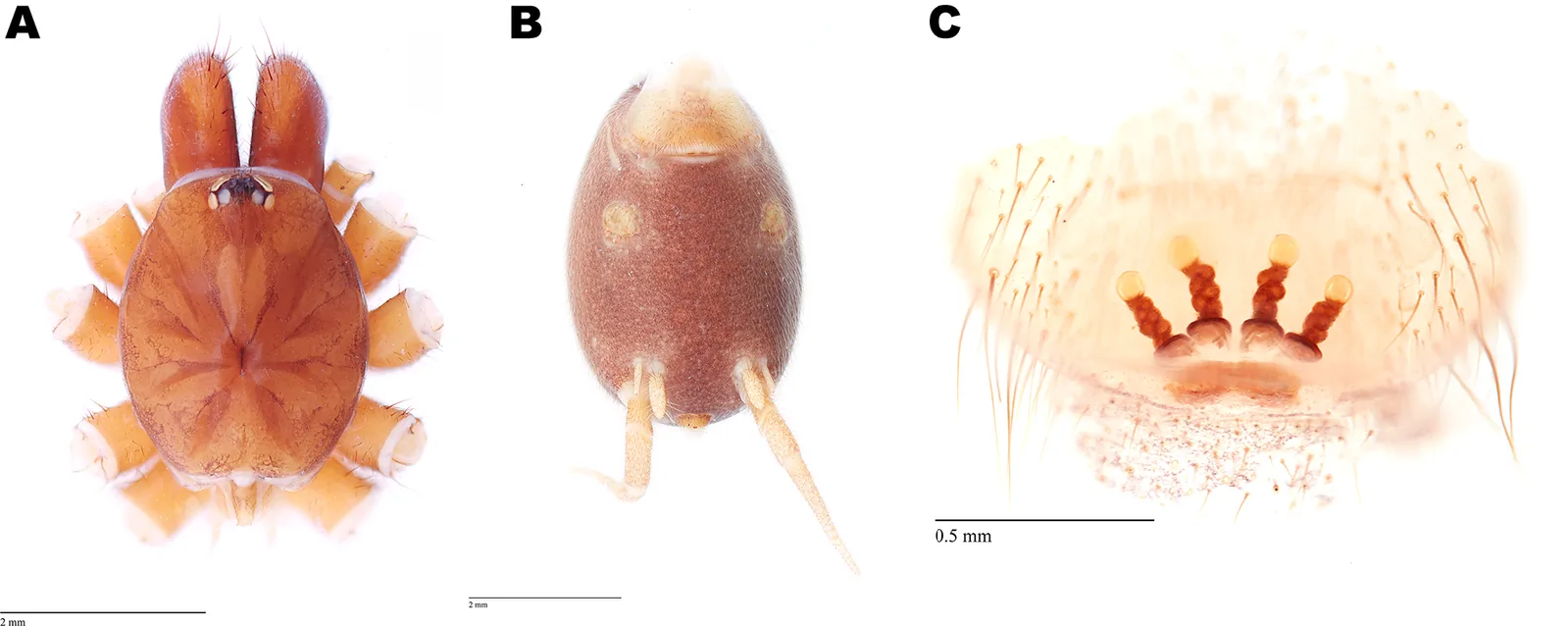
*Hexura picea*. **A**. ♀ cephalothorax, dorsal view (AMNH_IZC 00480902); **B**. ♀ abdomen, ventral view (AMNH_IZC 00480902); **C**. ♀ spermathecae (AMNH_IZC 00480908).

**Figure 6.**
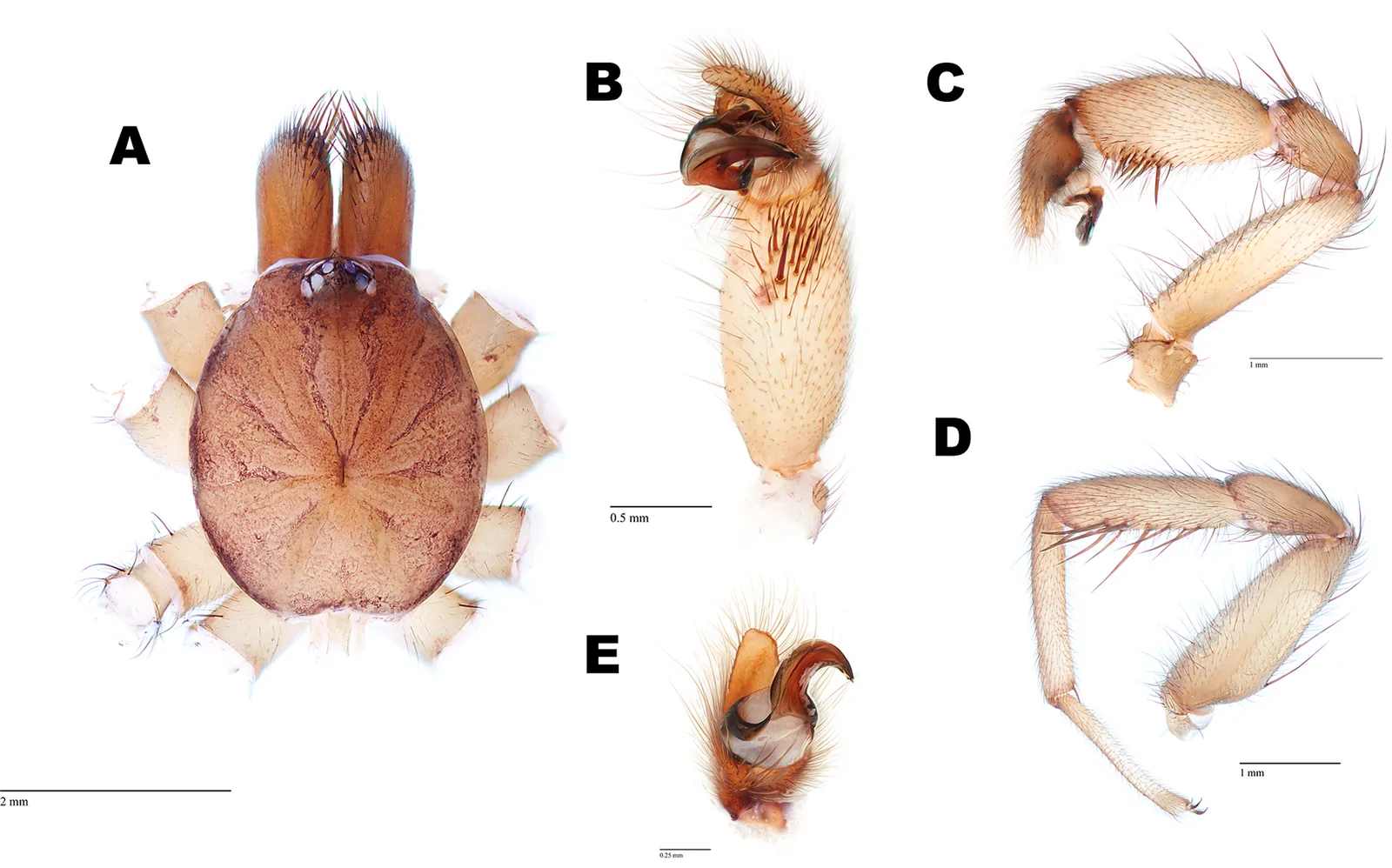
*Hexura picea* (HEX_009). **A**. ♂ cephalothorax, dorsal view; **B**. ♂ palpal tibia, ventral view; **C**. ♂ palp, retrolateral view; **D**. ♂ leg I, retrolateral view; **E**. ♂ palpal bulb, ventral view.

*Hexura picea* is easily differentiated from *H. rothi* by having three pairs of spinnerets instead of two and proportionally shorter chelicerae (Figs 5A, 5B, 6A). The tubular receptacles of female spermathecae are coiled and terminate in short, oval bulbs (Fig. 5C). Males of *H. picea* differ from *H. rothi* in having: 1) larger clasping spines on leg I tibia positioned more apically (Fig. 6D); 2) the presence of a ventral small spine patch beneath the apex of palpal tibia (Fig. 6B, C); 3) incrassate palpal tibia (Fig. 6C); and 4) palpal bulb with a heavy, curved conductor and fine embolus (Fig. 6B, C, E). Males of *H. picea* differ from *H. vandal* sp. nov. in having: 1) a denser number of clasping spines on leg I tibia (Fig. 6D); 2) a wider and shorter palpal tibia ventral spine patch (Fig. 6B, C); 3) an incrassate palpal tibia (Fig. 6C); and 4) a palpal bulb tip that is not bifurcated (Fig. 6B, C).

###### Distribution.

*Hexura picea* can be found in the Cascade Mountains in Washington and Oregon and the Coastal Ranges, from the Siuslaw National Forest to the southern parts of Vancouver Island in Canada (Fig. 1A).

###### Natural history.

*Hexura picea* is primarily associated with lowland to mid-elevation coniferous forests of the Pacific Northwest, typically occurring in moist forest floor microhabitats between approximately 0–500 m elevation, with occasional records extending into montane forests up to ~1300 m. They are commonly found in cool, moist microhabitats, such as moss mats, leaf litter, rotten logs or riparian forest floors, where they build their typical funnel web. Mature males have been collected from spring (April) to early autumn (November). This species is sometimes sympatric with the miniature mygalomorph funnel-web spider *Microhexura idahoana*, which shares very similar microhabitats.

###### Conservation status.

*Hexura picea* is widely distributed and commonly found throughout its distribution. In addition, the species is present in several protected areas such as Mt. Rainier National Park or Olympic National Park in Washington. Its conservation status is viewed as secure.

##### Hexura rothi

Taxon classificationAnimaliaAraneaeAntrodiaetidae

Gertsch & Platnick, 1979

07FC7B8C-EED1-52B7-A1C4-6A033BC8AE25

Figs 7, 8

Hexura rothi Gertsch & Platnick, 1979: 26, figs 64–66, 68–69 (male holotype and female paratype from McDonald Forest, north of Corvalis, Oregon, USA, in AMNH, New York, USA [examined]).

###### Material examined.

USA – Oregon, Curry Co: • 2 ♂, 2 juv. (AMNH IZC 00357960), Grants Pass (42.58389, −123.89528), 1150 m; 5 ♀, 4 juv. (AMNH IZC 00357963), Grants Pass (42.60167, −123.85639), 1250 m; 1 ♂ (AMNH IZC 00357964), Gold Beach, 13.x.1954, V. Roth leg. – Josephine Co: • 4 ♀, 5 juv. (AMNH IZC 00357961), Grants Pass (42.605, −123.78694), 1300 m; 3 ♀, 1 juv. (AMNH IZC 00357962), Cave Junction (42.19444, −123.40361), 900 m. – Benton Co: • 1 ♂, 1 ♀ (holotype + paratype) (AMNH IZC 00357965), McDonald Forest, Marys Peak, 29.ix.1959, V. Roth leg. – Marion Co: • 3 juv. (HEX_001–003), Silver Falls State Park (44.8619, −122.6380), 468 m, 15.viii.2024, A. Calatayud and E. Briggs leg.

###### Diagnosis.

**Figure 7.**
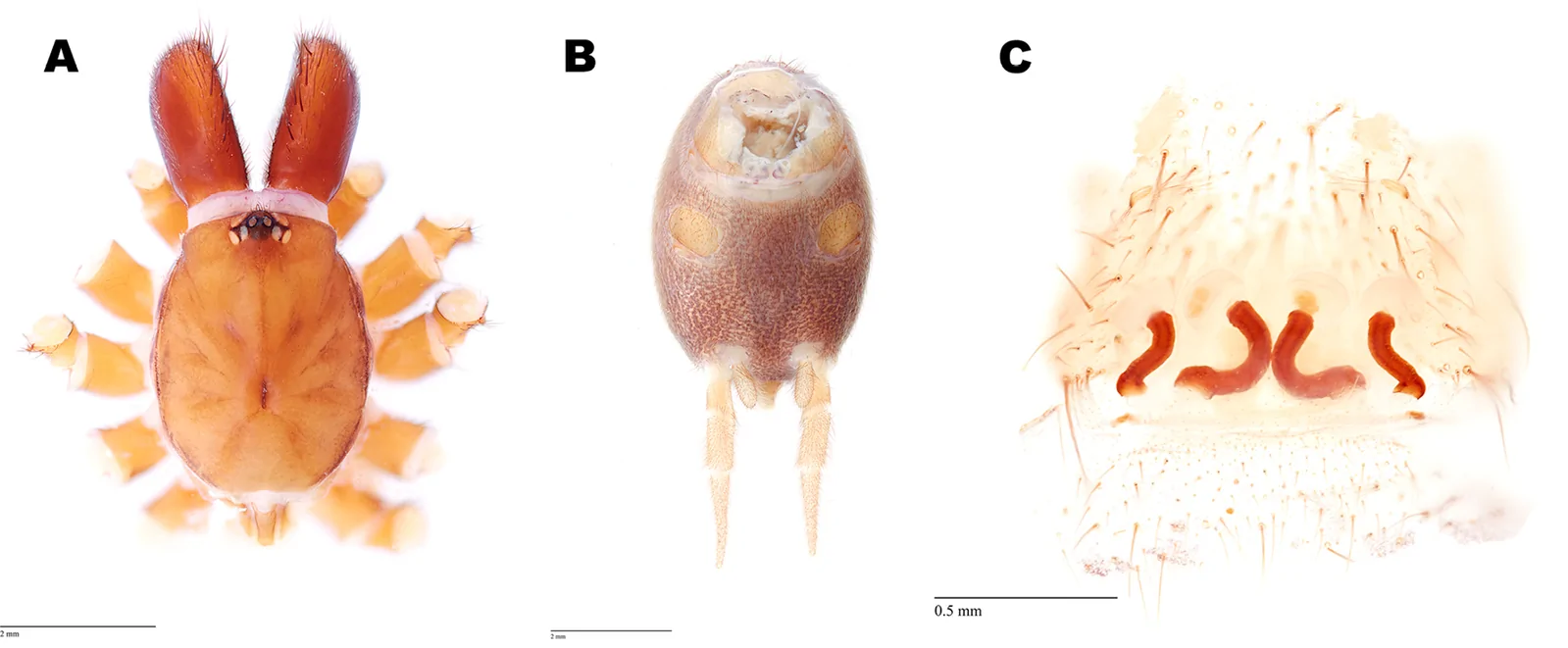
*Hexura rothi* (AMNH IZC 00357962). **A**. ♀ cephalothorax, dorsal view; **B**. ♀ abdomen, ventral view; **C**. ♀ spermathecae.

**Figure 8.**
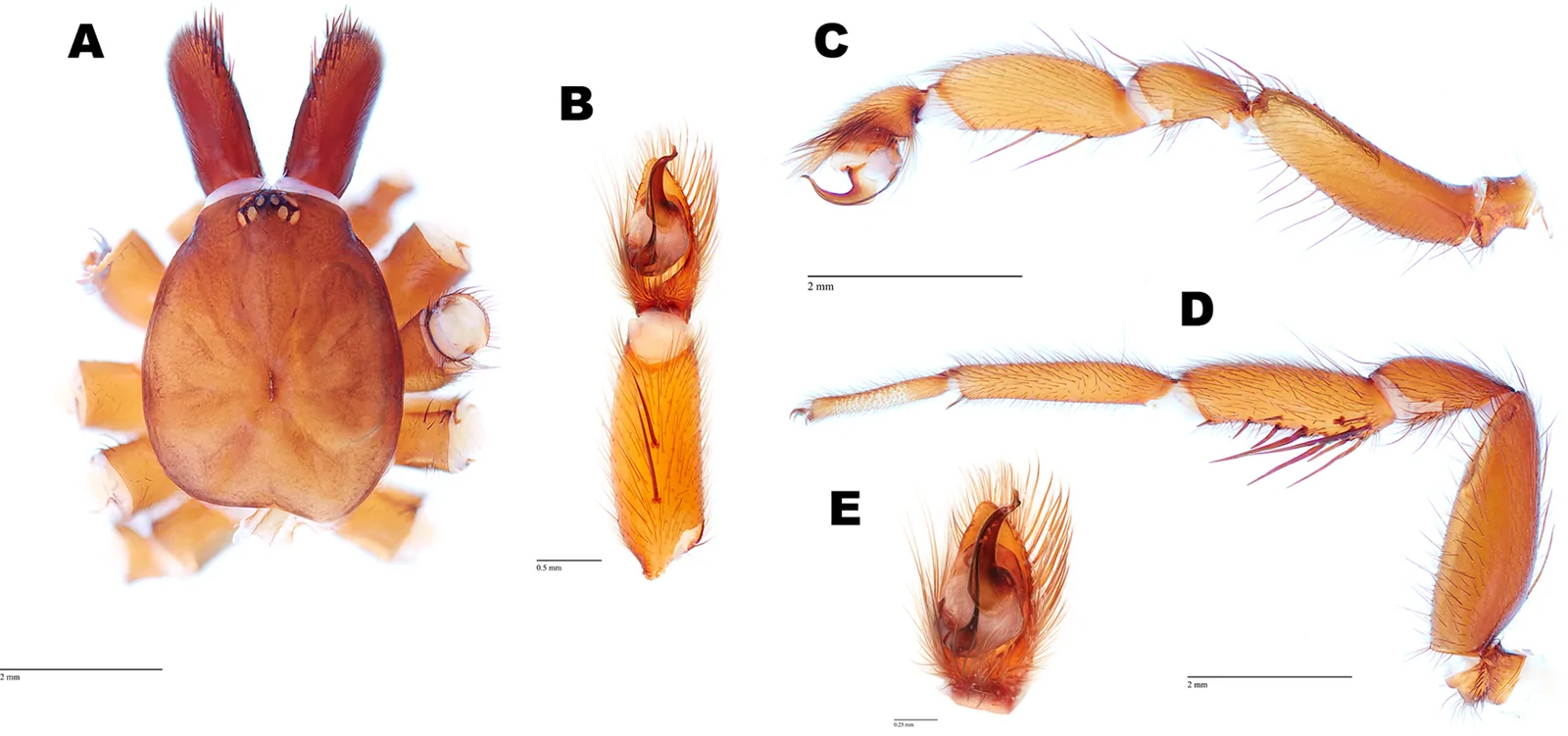
*Hexura rothi* (AMNH IZC 00357961). **A**. ♂ cephalothorax, dorsal view; **B**. ♂ palpal tibia, ventral view; **C**. ♂ palp, retrolateral view; **D**. ♂ leg I, retrolateral view; **E**. ♂ palpal bulb, ventral view.

*Hexura rothi* differs from the other species in the genus by possessing only two pairs of spinnerets, the anterior lateral pair being completely absent (Figs 7A, 7B, 8A). *Hexura rothi* can be further diagnosed by the tubular receptacles of female spermathecae being short, uncoiled and terminate in transparent, sub-oval pouches (Fig. 7C). Males of the species can be differentiated by 1) possessing larger clasping spines of leg I tibia clustered near the base (Fig. 8D); 2) the presence of only two large ventral spines on the palpal tibia (Fig. 8B, C); and 3) a palpal bulb with the embolus conductor longer and thin and twisted at the apex (Fig. 8B, C, E).

###### Distribution.

*Hexura rothi* is found in the Cascade Mountains and Coastal Ranges in southwestern Oregon (Fig. 1A).

###### Natural history.

*Hexura rothi* occupies a broad coastal–montane gradient in southwestern Oregon, ranging from near-sea-level coastal forests to lower montane conifer systems up to ~1200 m. They are commonly found in cool, moist microhabitats, such as rotten logs, where the entrance of their typical funnel web can be seen from the outside (Fig. 2C). Mature males have been collected in July, September, November and January.

###### Conservation status.

Although this species is less common and presents a narrower distribution range than the northern species, *H. picea*, its conservation status is likely secure due to its presence in protected areas throughout its distribution range such as Crater Lake National Park in Oregon.

##### Hexura vandal

Taxon classificationAnimaliaAraneaeAntrodiaetidae

Calatayud-Mascarell, Briggs & Hamilton sp. nov.

CB329210-A3BD-51E0-8FB2-8FEDE3FB8837

https://zoobank.org/4498F0C1-12D1-44E8-80E3-875D82078E32

Figs 9, 10, 11

###### Type material.

***Holotype***: USA – Idaho, Idaho Co • ♂ holotype; Apgar Creek, N of Campground, Kooskia (46.2143, −115.5385), 1050 m, 19.viii.2025, A. Calatayud-Mascarell and E. Briggs leg.; HEX_036; ***Paratype***: • ♀ paratype; 103 Rd, Weippe (46.3974, −115.6445), 1300 m, 7.ix.2025, A. Calatayud-Mascarell and C. Hamilton leg.; HEX_054.

**Figure 9.**
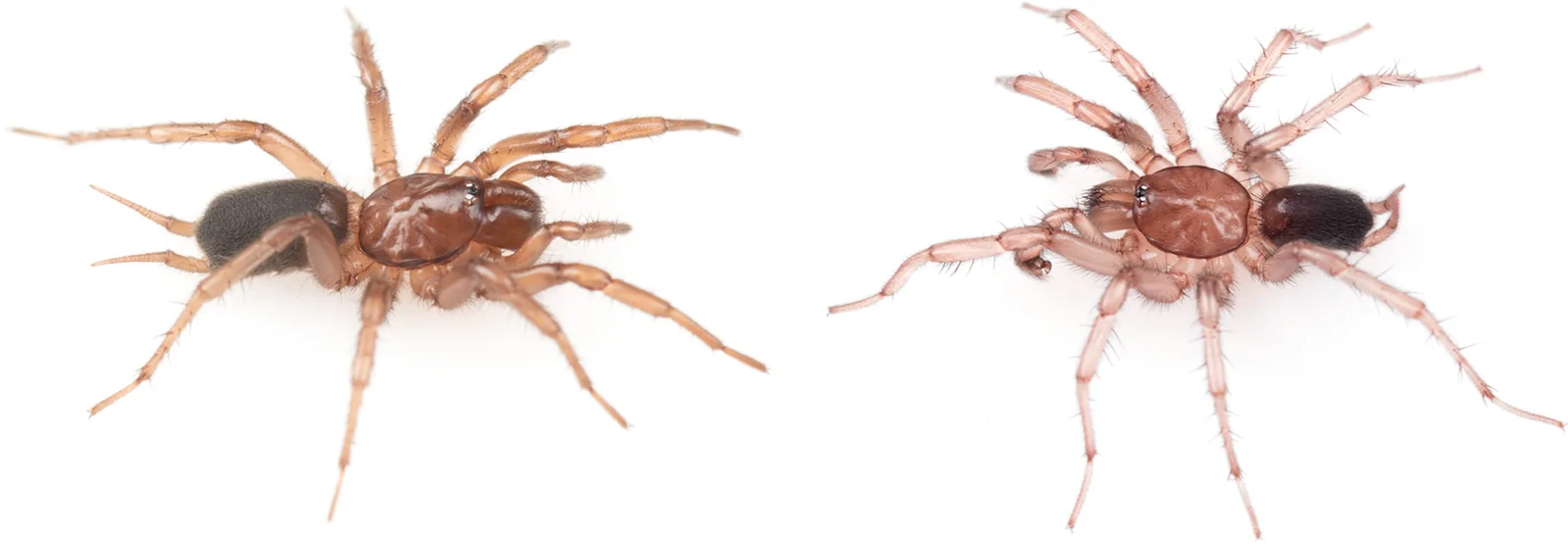
*Hexura vandal* sp. nov. female (L), male (R).

###### Material examined.

USA – Idaho, Idaho Co: • 4 ♀ (HEX_014–017), Apgar Creek, N of Campground, Kooskia (46.2143, −115.5385), 1050 m, 8.ix.2024, A. Calatayud-Mascarell and E. Briggs leg.; • 10 ♀, 2 juv. (HEX_018–024, 026–029, 031), same locality (46.2143, −115.5385), 1050 m, 2.v.2025, A. Calatayud-Mascarell and E. Briggs leg.; • 1 ♂, 4 ♀ (HEX_032–036), same locality (46.2143, −115.5385), 1050 m, 19.viii.2025, A. Calatayud-Mascarell and E. Briggs leg. – Clearwater Co: • 6 ♀, 2 juv. (HEX_037–044), Lolo Creek, 103 Rd (46.3768, −115.7195), 1200 m, 7.ix.2025, A. Calatayud-Mascarell and C. Hamilton leg.; • 10 ♀ (HEX_045–054), 103 Rd, Weippe (46.3974, −115.6445), 1300 m, 7.ix.2025, A. Calatayud-Mascarell and C. Hamilton leg.

###### Diagnosis.

**Figure 10.**
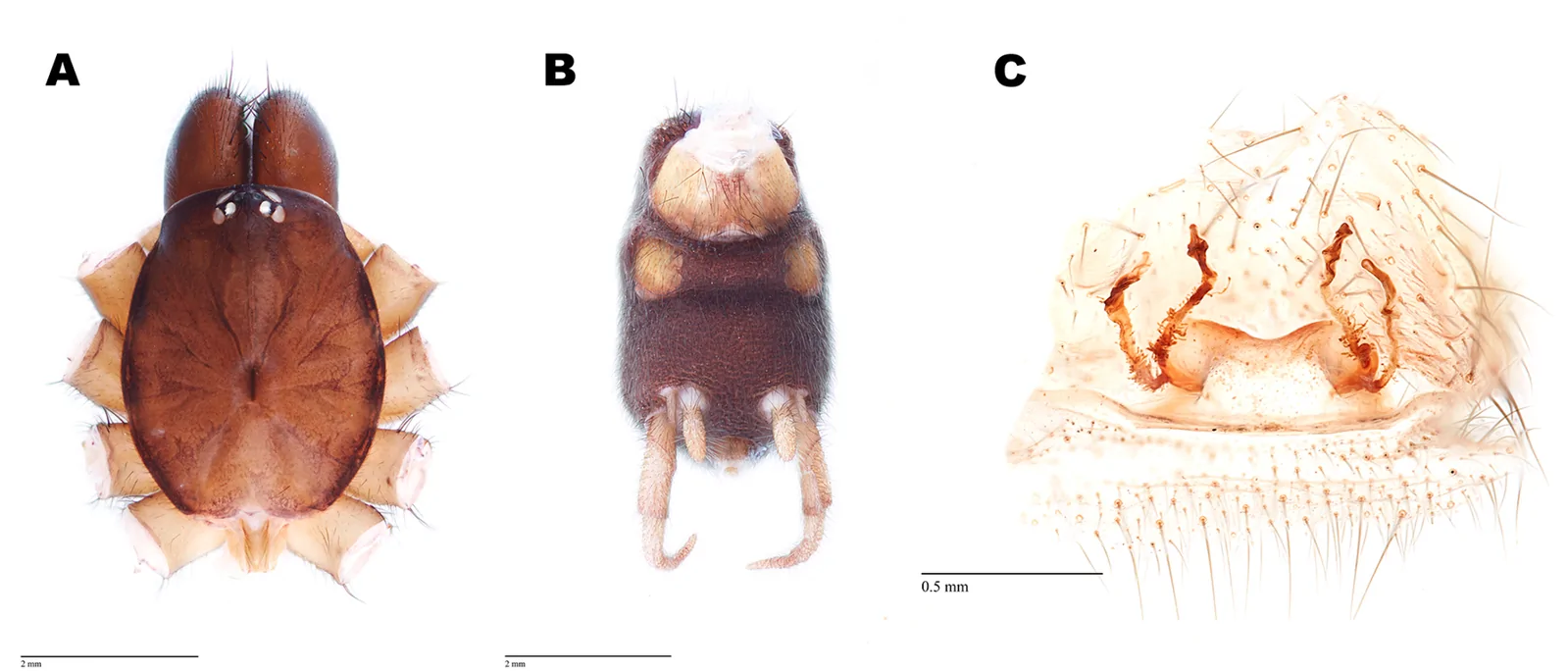
*Hexura vandal* sp. nov. **A**. ♀ cephalothorax, dorsal view (HEX_054); **B**. ♀ abdomen, ventral view (HEX_054); **C**. ♀ spermathecae (HEX_022).

**Figure 11.**
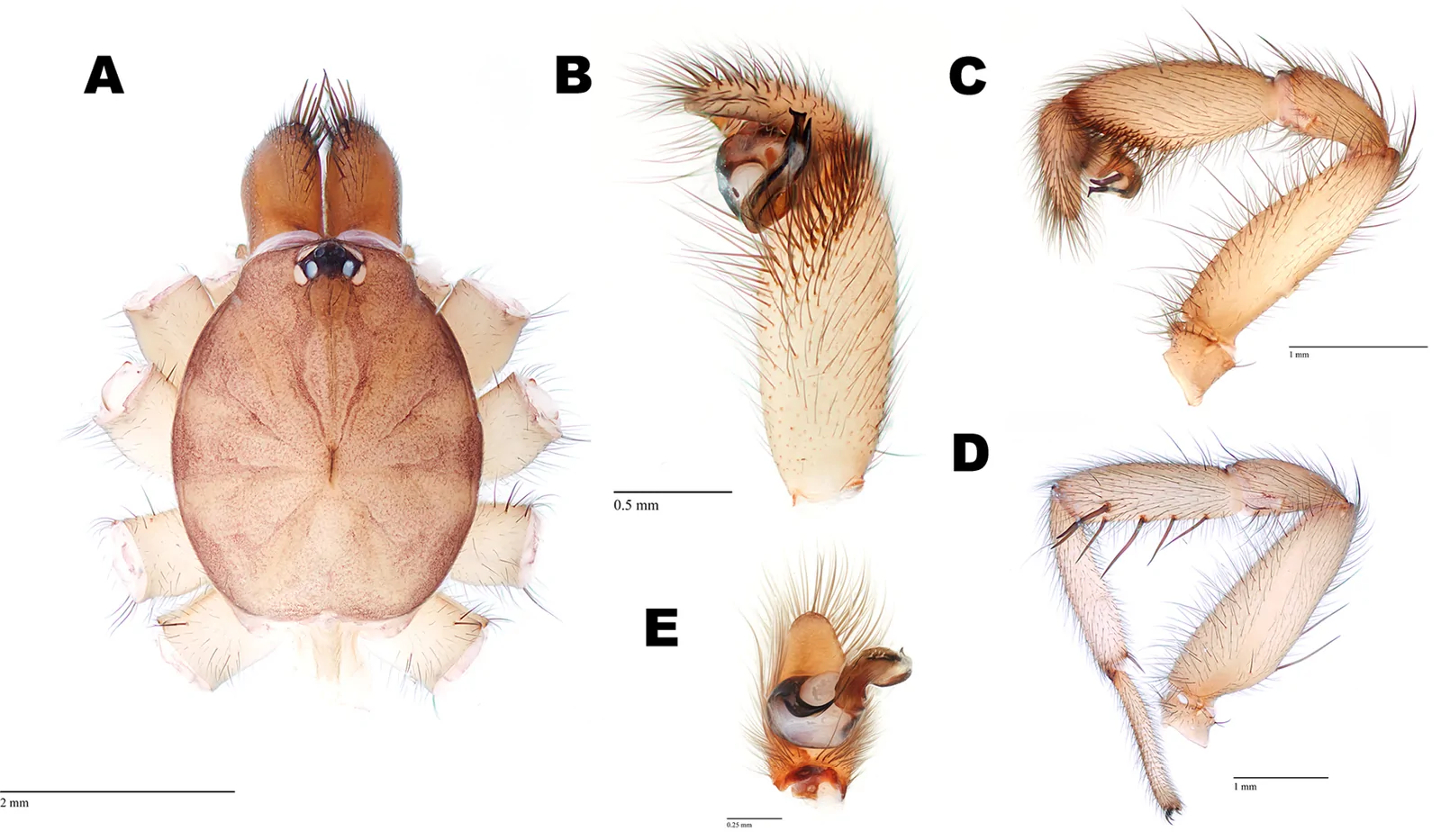
*Hexura vandal* sp. nov. (HEX_036). **A**. ♂ cephalothorax, dorsal view; **B**. ♂ palpal tibia, ventral view; **C**. ♂ palp, retrolateral view; **D**. ♂ leg I, retrolateral view; **E**. ♂ palpal bulb, ventral view.

*Hexura vandal* sp. nov. can be easily differentiated from *H. rothi* by possessing three pairs of spinnerets instead of two, and proportionally shorter chelicerae (Figs 10A, 10B, 11A). Females present a broad epigynal opening (Fig. 10B) and the tubular receptacles of the spermathecae are very thin, long, twisted with many small and short horizontal projections and terminate in rhomboidal bulbs (Fig. 10C). Males of *H. vandal* sp. nov. differ from *H. rothi* in having: 1) larger clasping spines on leg I tibia positioned more apically (Fig. 11D); 2) the presence of a ventral small spine patch beneath the apex of palpal tibia (Fig. 11B, C); and 3) a palpal bulb with a heavy, curved conductor and fine embolus bifurcated at the apex (Fig. 11B, C). Males of *H. vandal* sp. nov. differ from *H. picea* in having: 1) a smaller number of clasping spines on leg I tibia equally distributed (Fig. 11D); 2) a more narrow and longer palpal tibia ventral spine patch (Fig. 11B, C); and 3) a bifurcated palpal bulb tip (Fig. 11B, C, E).

###### Description of ♂ holotype.

(HEX_036; Fig. 11) Total length including chelicerae and spinnerets is 11.1 mm. Cephalothorax and chelicerae are light brown, with dark streaks from side margins running into the fovea. Appendages, spinnerets, and sternum are yellow cream. Abdomen dark brown with lighter abdominal tergites that cover almost the entire width and one-fourth of the length of the abdomen. Specimens look darker alive. Carapace is 3.2 mm long and 2.7 mm wide, glabrous, suboval dorsally with a rectangular projection at the eyes and eight subtle cephalic grooves, convex laterally. Fovea small and straight. Eyes set on small tubercle one-third width of anterior carapace. Clypeus short. Eyes: AME 0.14, PME 0.08, ALE 0.18, PLE 0.24; AME-AME 0.32, PME-PME 0.18, ALE-ALE 0.43, PLE-PLE 0.54, ALE-AME 0.11, ALE-PLE 0.23, AME-PLE 0.18, AME-PME 0.16, PME-PLE 0.12. Sternum 2.1 mm long, 1.52 mm wide, sparsely covered with hairs concentrated on lateral edges, sternal sigilla not obvious. Labium 0.51 mm long, 0.54 mm wide, with forward-projecting hairs. Endites 1.1 mm long, 0.62 mm wide. Chelicerae 1.3 mm long, 0.67 mm wide with a dense row of long spines on the internal margin, promargin with single row of 11 pale yellow teeth, black at the tip, with enlarged teeth near base fang. Leg formula 4213. Legs proportionally longer, thinner, with fewer spines on legs I and II; tibia I with seven evenly spaced stout spines ventrally. Leg measurements: Leg I: femur 2.7, width 0.94, patella 1.2, tibia 2.0, metatarsus 1.8, tarsus 1.6. Leg II: femur 2.6, width 0.9, patella 1.1, tibia 1.9, metatarsus 2.2, tarsus 1.9. Leg III: femur 2.5, width 0.8, patella 1.0, tibia 2.0, metatarsus 2.5, tarsus 1.8. Leg IV: femur 2.9, width 0.8, patella 1.2, tibia 2.6, metatarsus 3.3, tarsus 2.2. Palpal tibia 1.6 long, 0.7 wide. Palp with a heavy, curved conductor and fine embolus bifurcated at the apex, tibia with a long and narrow cluster of small spines beneath at apex. Abdomen 3.2 mm long, 2.2 mm wide, suboval. Posterior median spinnerets cylindrical, half the size of the anterior laterals, conical; posterior lateral spinnerets 3.8 mm long, tapering, three-segmented.

###### Description of ♀ paratype.

(HEX_054; Fig. 10) Total length including chelicerae and spinnerets is 13.1 mm. Coloration and structure essentially as in male. Carapace 3.7 long and 3.1 wide, glabrous, oval dorsally. Eyes: AME 0.15, PME 0.09, ALE 0.20, PLE 0.27; AME-AME 0.41, PME-PME 0.22, ALE-ALE 0.71, PLE-PLE 0.55, ALE-AME 0.15, ALE-PLE 0.27, AME-PLE 0.20, AME-PME 0.16, PME-PLE 0.14. Sternum 2.0 mm long, 1.7 mm wide, sparsely covered with hairs concentrated on lateral edges, with two sternal sigilla aligned with leg III coxa. Labium 0.67 mm long, 0.62 mm wide, with forward-projecting hairs. Endites 1.3 mm long, 0.85 mm wide. Chelicerae 1.4 mm long 1.0 mm wide with a row of a few long spines on the internal margin, promargin with nine small black teeth in single row, with enlarged teeth near base fang. Leg measurements. Leg I: femur 2.7, width 1.1, patella 1.2, tibia 1.9, metatarsus 1.7, tarsus 1.3. Leg II: femur 2.6, width 1.0, patella 1.2, tibia 1.7, metatarsus 1.7, tarsus 1.3. Leg III: femur 2.4, width 0.9, patella 1.0, tibia 1.7, metatarsus 2.1, tarsus 1.4. Leg IV: femur 2.9, width 0.9, patella 1.1, tibia 2.3, metatarsus 2.8, tarsus 1.4. Abdomen 4.6 mm long, 2.6 mm wide, suboval, with a broad epigynal opening. Spinnerets as in males. Spermathecae with the tubular receptacles of epigynum very thin, long, twisted with many small and short horizontal projections and terminate in rhomboidal bulbs.

###### Distribution.

Known only from the type locality near Kooskia, Idaho and surroundings localities in the Nez Perce National Forest north of the Lochsa River (Fig. 1).

###### Natural history.

*Hexura vandal* sp. nov. inhabits mid-elevation (1050–1300 m) mixed conifer forests with a dense fern understory. The microhabitats are found in cool, moist environments such as north-facing slopes, riparian zones, or shaded canyons. *H. vandal* sp. nov. build a dense funnel web inside or under rotten logs, or in the moss mat. The entrance of the funnel web can usually be seen from outside of their retreat. Males mature during summer (August, *N* = 2). Pedipalps of immature males are swollen, and they can molt several times before reaching maturity. Three females kept in captivity produced egg sacs in late May, a few weeks after collection, suggesting that spiderlings likely hatch during spring. Unfortunately, none of the egg sacs were viable.

###### Etymology.

The specific epithet is a noun in apposition referring to the nickname “Vandals” adopted by the University of Idaho in 1917 after a student journalist, Harry Lloyd McCarty, described the men’s basketball team’s aggressive defense as “vandalizing” their opponents. Idaho is the only Division I university with this mascot, and this spider species is endemic to Idaho. The name honors the university, which provided partial funding for this study, and represents how the University of Idaho is the “gem” of the Gem State’s higher education system.

###### Conservation status.

*Hexura vandal* sp. nov. occupies a very restricted distribution. Multiple sampling campaigns radiating out of its known distribution were not successful, limiting its known distribution to the type locality and the other two close localities. However, the area is fairly remote and is limited by forest roads that make it difficult to conduct comprehensive sampling throughout the region. Human activities such as logging are common throughout the area, representing a potential threat for the species, especially due to the lack of protected habitat. Wildfires are common in this region and increasing yearly. Additionally, wildfire season occurs during the mating season, resulting in a significant threat for the species in the future. Due to the combination of a small distribution range and its potential threats for future survival, the conservation status of *H. vandal* sp. nov. is threatened and likely endangered.

## Supplementary Material

XML Treatment for Hexura picea

XML Treatment for Hexura rothi

XML Treatment for Hexura vandal

## Additional information

### Artificial Intelligence (AI) use

The authors accept full responsibility for the content of the manuscript, including the disclosure of any use of AI.

No AI tools were used in the preparation of this manuscript.

### Author ORCIDs

A. Calatayud-Mascarell https://orcid.org/0000-0003-0627-2616

E.J. Briggs https://orcid.org/0000-0002-3475-5669

C.A. Hamilton https://orcid.org/0000-0001-7263-0755

### Data availability

All of the data that support the findings of this study are available in the main text or Supplementary Information.

## Supplementary materials

Supplementary material 1 Supplementary figures Data type pdf Explanation note **fig. S1**. Species tree using ASTRAL-III. **fig. S2**. Morphometric results of linear measurements. https://binary.pensoft.net/file/1758704This dataset is made available under the Open Database License (http://opendatacommons.org/licenses/odbl/1.0/). The Open Database License (ODbL) is a license agreement intended to allow users to freely share, modify, and use this Dataset while maintaining this same freedom for others, provided that the original source and author(s) are credited. Arnau Calatayud-Mascarell, Ethan J. Briggs, Chris A. Hamilton

Supplementary material 2 Specimen data Data type xlsx https://binary.pensoft.net/file/1758705This dataset is made available under the Open Database License (http://opendatacommons.org/licenses/odbl/1.0/). The Open Database License (ODbL) is a license agreement intended to allow users to freely share, modify, and use this Dataset while maintaining this same freedom for others, provided that the original source and author(s) are credited. Arnau Calatayud-Mascarell, Ethan J. Briggs, Chris A. Hamilton
