# Supplementary material for: Hidden in the woods: A new species of Hexura (Araneae, Mygalomorphae, Antrodiaetidae) from a highly restricted range in Idaho
Source: Zookeys. 2026 Aug 31;1290:339–55. doi: 10.3897/zookeys.1290.200898 (PMC13551060; doi:10.3897/zookeys.1290.200898)
Supplement: Supplementary file 1 [file zookeys-1290-339_article-200898__-s001.pdf]

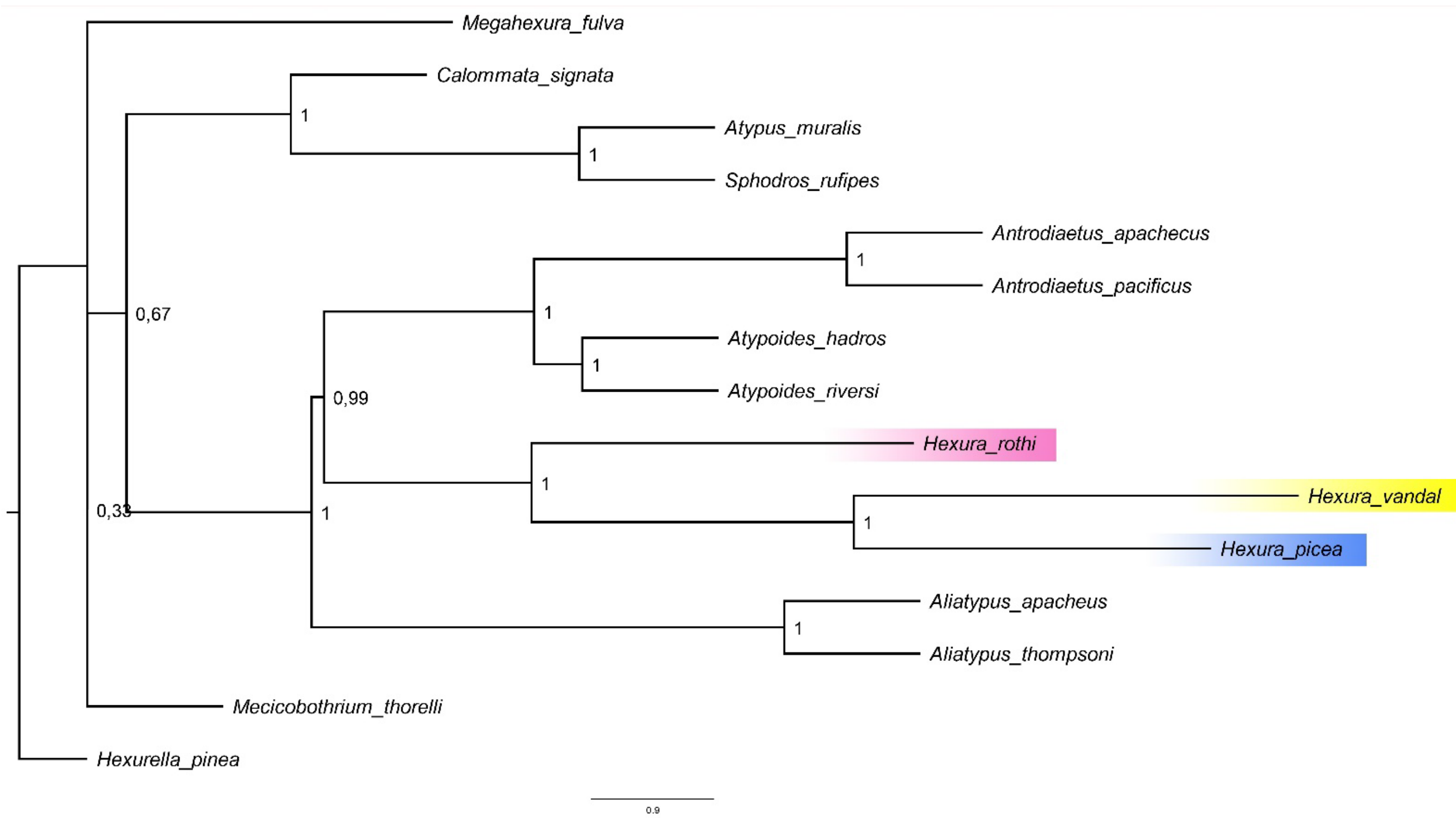

figure S1. Species tree using ASTRAL-III.

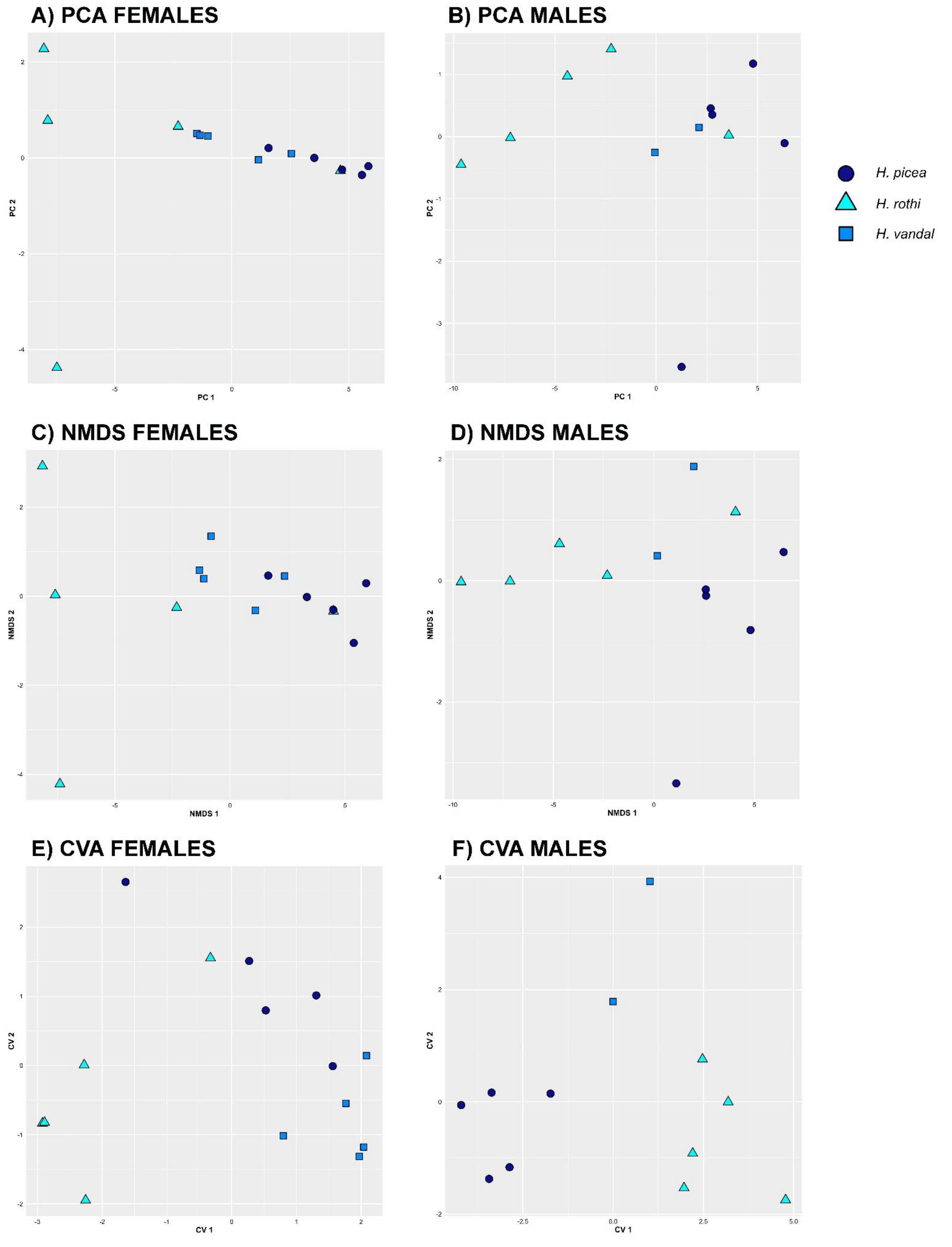

figure S2. Morphometric results of linear measurements. **A.** Females PCA; **B.** Males PCA; **C.** Females NMDS; **D.** Males NMDS; **E.** Females CVA; **F.** Males CVA.
